# Low-temperature leaf photosynthesis of a *Miscanthus* germplasm collection correlates positively to shoot growth rate and specific leaf area

**DOI:** 10.1093/aob/mcw042

**Published:** 2016-05-13

**Authors:** Xiurong Jiao, Kirsten Kørup, Mathias Neumann Andersen, Karen Koefoed Petersen, Thomas Prade, Stanisław Jeżowski, Szymon Ornatowski, Barbara Górynowicz, Idan Spitz, Poul Erik Lærke, Uffe Jørgensen

**Affiliations:** ^1^Aarhus University, Department of Agroecology, Blichers Allé 20, DK-8830 Tjele, Denmark,; ^2^Aarhus University, Department of Food Science, Kirstinebjergvej 10, DK-5792 Aarslev, Denmark,; ^3^Swedish University of Agricultural Sciences, Department of Biosystems and Technology, SE-230 53 Alnarp, Sweden,; ^4^Institute of Plant Genetics, Polish Academy of Sciences, Strzeszyńska 34, 60-479 Poznań, Poland and; ^5^University of Illinois, Institute of Genomic Biology, 1206 W. Gregory Dr. 138 IGB, Urbana, IL 61801, USA

**Keywords:** Cold tolerance, C_4_ photosynthesis, dark-adapted chlorophyll fluorescence, genotypic difference, light and CO_2_ response curves, *M. sacchariflorus*, *M. sinensis*, *M. tinctorius*, *M. × giganteus*

## Abstract

**Background and Aims** The C_4_ perennial grass miscanthus has been found to be less sensitive to cold than most other C_4_ species, but still emerges later in spring than C_3_ species. Genotypic differences in miscanthus were investigated to identify genotypes with a high cold tolerance at low temperatures and quick recovery upon rising temperatures to enable them to exploit the early growing season in maritime cold climates. Suitable methods for field screening of cold tolerance in miscanthus were also identified.

**Methods** Fourteen genotypes of *M. sacchariflorus*, *M. sinensis*, *M. tinctorius* and *M.* × *giganteus* were selected and grown under warm (24 °C) and cold (14 °C) conditions in a controlled environment. Dark-adapted chlorophyll fluorescence, specific leaf area (SLA) and net photosynthetic rate at a photosynthetically active radiation (PAR) of 1000 μmol m^–2^ s^–1^ (*A*_1000_) were measured. Photosynthetic light and CO_2_ response curves were obtained from 11 of the genotypes, and shoot growth rate was measured under field conditions.

**Key Results** A positive linear relationship was found between SLA and light-saturated photosynthesis (*A*_sat_) across genotypes, and also between shoot growth rate under cool field conditions and *A*_1000_ at 14 °C in a climate chamber. When lowering the temperature from 24 to 14 °C, one *M. sacchariflorus* exhibited significantly higher *A*_sat_ and maximum photosynthetic rate in the CO_2_ response curve (*V*_max_) than other genotypes at 14 °C, except *M*. × *giganteus* ‘Hornum’. Several genotypes returned to their pre-chilling *A*_1000_ values when the temperature was increased to 24 °C after 24 d growth at 14 °C.

**Conclusions** One *M. sacchariflorus* genotype had similar or higher photosynthetic capacity than *M*. × *giganteus*, and may be used for cultivation together with *M*. × *giganteus* or for breeding new interspecies hybrids with improved traits for temperate climates. Two easily measured variables, SLA and shoot growth rate, may be useful for genotype screening of productivity and cold tolerance.

## INTRODUCTION

C_4_ plant species are considered to have a carbon assimilation advantage over C_3_ species because of their ability to concentrate CO_2_ around Rubisco in the bundle sheath cells, thus reducing the risk of photo-oxidation. C_4_ species dominate in warm and light-intensive environments, while they are rare in cool climates because of a marked loss of photosynthetic capacity at low temperatures (Long, 1999), and the distribution of most C_4_ species is limited by a mean minimum temperature of 8–10 °C during the period of active growth (Long, 1983). However, of all the C_4_ plants, the perennial rhizomatous C_4_ grass miscanthus (*M.* × *giganteus*) exhibits exceptional cold tolerance (Beale and Long, 1995; Dohleman and Long, 2009). Because of its high yield potential under cool climatic conditions and rapid accumulation of biomass, it has been proposed as a potential bioenergy crop in northern Europe and the USA (Heaton *et al.*, 2008; Zub and Brancourt-Hulmel, 2010; Long and Spence, 2013).

Previous studies have mainly focused on the sterile triploid hybrid *M.* × *giganteus*, which has been reported to have a high photosynthetic capacity (Wang *et al.*, 2008*a*, *b*), leaf expansion rate (Clifton-Brown and Jones, 1997) and biomass productivity in central and northern Europe at mean annual temperatures of 7·3–8·0 °C (Lewandowski *et al.*, 2000).

*Miscanthus* × *giganteus* is a natural sterile hybrid between *M. sacchariflorus* and *M. sinensis*, which was introduced into Europe in the 1930s and has been widely investigated and grown for bioenergy (Greef and Deuter, 1993). The original clone is now widely distributed across Europe and the USA, where many local varieties have been named. However, different varieties of *M.* × *giganteus* are genetically very similar (Głowacka *et al.*, 2015*a*). The genus *Miscanthus* has its origin in the tropics and sub-tropics, but different species are found throughout a wide climatic range in East Asia (Greef and Deuter, 1993). Gauder *et al.* (2012) found that *M.* × *giganteus* produced higher yields than clones of *M. sacchariflorus*, *M. sinensis* and *M. sinensis* hybrids in a field study in south-western Germany. However, in newly established miscanthus plantations, several *M. sinensis* genotypes are reported to have greater frost tolerance and winter survival than *M.* × *giganteus* and *M. sacchariflorus* (Clifton-Brown and Lewandowski, 2000). Investigations within different miscanthus species and environments of origin may therefore help to broaden the genetic base for the photosynthetic response to low temperatures which is useful when breeding for new high-yielding hybrids with improved cold tolerance (Głowacka *et al.*, 2014). The availability of a larger number of high-quality genotypes might also increase the resistance to pests and diseases in large-scale plantations.

*Miscanthus* × *giganteus* has previously been found to form photosynthetically competent leaves at lower temperatures than maize (Naidu, 2003) and to have a larger leaf area and longer growing season than maize (Dohleman and Long, 2009). The reason for the ability of *M.* × *giganteus* to maintain photosynthetic capacity at low temperatures is apparently related to a higher content and activity of pyruvate orthophosphate dikinase (PPDK) (Naidu *et al.*, 2003; Wang *et al.*, 2008*b*) and Rubisco (Spence, 2012), and not of phosphoenolpyruvate carboxylase (PEPc) (Naidu and Long, 2004). A higher content of the carotenoid zeaxanthin may also help to protect photosystem II (PSII) from photoinhibition (Farage *et al.*, 2006). Although miscanthus emerges later in spring and thus has a delayed canopy development compared with C_3_ grass species, recent studies indicate that the limitations due to temperature are not an inherent C_4_ trait that cannot be circumvented (Long and Spence, 2013). This increases the feasibility of finding genotypes that are better suited to temperate areas and can better utilize the entire length of the growing season in cool climates.

Even though *M.* × *giganteus* is relatively cold tolerant, it is likely that further cold tolerance exists within the *Miscanthus* genus (Głowacka *et al.*, 2015*b*). To utilize the whole growing season fully, quick recovery of photosynthesis when exposed to higher temperatures is another important feature that plants should possess when grown in maritime climates. Thus, it will be helpful for future breeding work if miscanthus genotypes had a high cold tolerance at low temperatures in conjunction with a rapid restorative capacity of the photosynthesis when temperatures rise. Another essential variable to consider is the canopy development in early spring. Efficient capture of solar radiation in early spring is dependent not only on cold-tolerant photosynthetic processes, but also on fast leaf area development. At the canopy level, a large specific leaf area (SLA) allows a more extensive foliar display for a similar biomass investment in leaves, resulting in improved light absorption (Niinemets, 1998). In miscanthus, nitrogen fertilization did not increase the net photosynthetic rate (*A*), but increased SLA (Wang *et al.*, 2012), which means that thin leaves at high N-fertilization rates resulted in similar values of *A* but increased the photosynthetic rate per gram of leaf (*A*_mass_). Such a positive increase in *A*_mass_ with N-fertilization has also been observed in willow (*Salix viminalis* and *Salix dasyclados*) (Merilo *et al.*, 2006). It would therefore be interesting to investigate whether the genetic variation within a species would also code for differences in resource investment in leaves for photosynthesis, and if these differences are reflected in different *A*_mass_.

In previous investigations, the leaf elongation rate has been used to compare miscanthus (Purdy *et al.*, 2013) and maize (Reymond *et al.*, 2003) genotypes at low temperatures. However, the elongation rate depends on the developmental stage of the leaf (Durand *et al.*, 1999), and it is difficult to compare between genotypes due to the requirement for exactly the same age of the leaf (Muller *et al.*, 2001). It may thus be more promising to compare shoot growth rates during early vegetative stages of miscanthus growth (Clifton-Brown and Jones, 1997).

Here we studied 14 miscanthus genotypes selected from a collection of 166 genotypes on the basis of shoot growth rate, and we tested for its correlation with assimilation rate. We compared the photosynthetic performance under warm and subsequent cool conditions, and also determined whether they were able to recover their photosynthetic capacity fully upon return to warm conditions. Our hypothesis was that we would be able to identify genotypes with improved photosynthetic performance under cold conditions and with a rapid recovery rate during a temperature rise. In addition, we expected that genotypes with higher SLA and a stable *A* could be selected.

## MATERIALS AND METHODS

### Plant material

A total of 14 genotypes were selected for a detailed climate chamber experiment. Four species of miscanthus, namely *M. sinensis*, *M. sacchariflorus*, *M. tinctorius* and *M.* × *giganteus* (*M. sinensis* × *M. sacchariflorus*), were represented (Table 1). They were selected from a pool of 166 genotypes screened under field conditions (Supplementary Data Fig. S1) based on their shoot growth rate and leaf gas exchange rate in 2012, as described below. Of the 166 screened genotypes, 27 were grown in Poland (Institute of Plant Genetics of the Polish Academy of Sciences, Poznań, 50°2'N, 21°59'E), 41 in Sweden (Department of Biosystems and Technology, Swedish University of Agricultural Sciences, Alnarp, 55°39'N, 13°05'E) and 98 in Denmark (Department of Agroecology, Aarhus University, Foulumgaard, 56°30'N, 9°35'E). Eleven genotypes were selected from the Danish collection, of which three originated from the European Miscanthus Improvement (EMI) project (Lewandowski *et al.*, 2000; Clifton-Brown *et al.*, 2001; Jørgensen *et al.*, 2003) and eight were collected on Honshu and Hokkaido Islands in Japan in October 1995 (Kjeldsen *et al.*, 1999). The reference *M.* × *giganteus* was the clone ‘Hornum’ (Głowacka *et al.*, 2015*a*) which was grown for many years at Hornum in Jutland, Denmark (Jørgensen, 1997), and was confirmed as a sterile triploid (Linde-Laursen, 1993). The genotype Sin-7 was selected from the Swedish collection, and Sin-6 and Gig-14 (a hybrid bred at Tinplant, Germany) from the Polish collection. The share of the gene pool screened in Denmark was a part of the larger pool previously screened for cold tolerance by chlorophyll fluorescence (*F*_v_/*F*_m_) in a separate study (Głowacka *et al.*, 2015*b*). Only three genotypes (Tin-1, Sin-3 and Sac-10) were used in both the study of Glowacka *et al.* and our study.

**Table 1.** Genotypes measured in the climate chamber experiments

Identification	Species	Origin	Altitude (m)	Latitude (ºN)	Ploidy
Tin-1	*M. tinctorius*	Ainukura, Honshu*	350	36	2*x*
Sin-2	*M. sinensis*	Yoichi, Hokkaido; OUAV collection*		43	2*x*
Sin-3	*M. sinensis*	Kamiyoshino (5 km south-east of Kanazawa), Honshu*	280	36	2*x*
Sin-4	*M. sinensis*	North-east of Obihiro, Hokkaido*		43	2*x*
Sin-5	*M. sinensis*	South of Shirakawa, Honshu*	600	36	2*x*
Sin-6	*M. sinensis*	Tinplant GmbH, Klein Wanzleben, Germany			2*x*^†^
Sin-7	*M. sinensis*	Hokkaido		43	2*x*
Sin-H8	*M. sinensis*	Hybrid from Deuter, Germany^‡^			3*x*^‡^
Sac-9	*M. sacchariflorus*	Biratori (south-east of Sapporo), Hokkaido*		42	4*x*
Sac-10	*M. sacchariflorus*	Hakusan National Park, Honshu*	900	36	4*x*
Sac-11	*M. sacchariflorus*	South of Shirakawa, Honshu*	600	36	4*x*
Sac-12	*M. sacchariflorus*	Tinplant GmbH, Klein Wanzleben, Germany^‡^			4*x*^‡^
Gig-13	*M.×giganteus*	Cultivar Hornum^§^, Larsen, Denmark^‡^			3*x*^‡^
Gig-14	*M.×giganteus*	Tinplant, GmbH, Klein Wanzleben, Germany^¶^			2x**

^*^Seed plants collected in Japan 1995 and grown in Denmark since 1996 (Kjeldsen *et al*., 1999).

^†^Ploidy level was analysed at the Institute of Plant Genetics, Poland.

^‡^Clones from the ‘European *Miscanthus* Improvement’ project; Sac-12 corresponds to EMI-5 and Sin-H8 to EMI Sin-H6 reported by Clifton-Brown *et al*. (2001).

^§^EMI-1 was equivalent to Hornum, since they are almost genetically similar (Głowacka *et al*., 2015*a*).

^¶^Gig-14 corresponds to M114 reported by Sampson *et al*., (2012), origin information is reported in Kim *et al*. (2012).

^**^Martin Deuter, pers. comm.

### Ploidy examination

The ploidy levels of genotypes Tin-1, Sin-2, Sin-3, Sin-4, Sin-5, Sac-9, Sac-10 and Sac-11 were not known from the literature and therefore were determined by flow cytometry. Flow cytometry was applied to leaves or rhizomes by a Partec PA II flow cytometer equipped with an HBO-100 mercury arc lamp (Partec GmbH, Germany) and a filter combination for 4',6-diaminino-2-phenylindole (DAPI) staining (Partec 06-03-310). Samples were chopped for 30 s in a Petri dish containing 0·6 mL of citric acid buffer and left for 5 min to allow release of nuclei. The nuclei were stained by adding 2·5 mL of fluorescent solution containing 5 μM DAPI and left for another 5 min (Otto, 1990). The suspension of nuclei was passed through a nylon filter with pore size of 50 μm to remove large debris. The relative fluorescence of the total DNA of single nuclei was analysed, and in each sample the DNA content of 5000 nuclei was checked. Samples of a diploid *M. sinensis* genotype (MS 88-110) were used as an internal standard.

### Screening in the field by determination of shoot growth rate

The leaf elongation rate is difficult to compare between genotypes (Muller *et al.*, 2001), so instead we chose to measure the daily shoot growth rate, which is defined as the difference in the distance between the soil surface and the tip of the tallest leaf measured on two different days, divided by the number of days between the measurements.

In May 2012, five shoots from all genotypes were marked. Using a transparent ruler (IMPEGA) with a precision of 1 mm, shoot length was measured every second or third day from shoot emergence. From these measurements, the average daily growth rate was calculated for a cold and a warm period as described below. The temperature was measured 20 cm above the soil surface in the field. In Denmark a cold period lasted from 30 May until 1 June with a daily mean temperature of 9·8 ± 0·7°C. This cold period succeeded a warm period from the 24 to 26 May, with an average temperature of 18·8 ± 0·2°C. The cool and warm periods for Sweden, determined by the daily mean temperatures, were from 4 to 7 May (9·7 ± 0· 9°C) and from 9 to 11 May (14·8 ± 0·4°C), respectively, and for Poland from 14 to 16 May (10·5°C) and from 9 to 11 May (24°C), respectively.

### Screening in the field by gas exchange measurements

A total of 37 genotypes that displayed the most vigorous growth in the first 2 weeks of May 2012 were selected for gas exchange measurements in the field with 2, 3 or 4 d intervals from 18 May until 4 June 2012. The measurements were performed using CIRAS-2 (PP Systems, Amesbury, MA, USA) equipment on the same leaves as those measured for shoot growth rate. The leaves were dry and were measured between the hours of 1000 and 1400, with the following cuvette chamber conditions: photosynthetic active radiation (PAR) of 1600 μmol m^–2^ s^–1^, CO_2_ concentration of 390 μmol mol^–1^ and relative humidity (RH) as close to ambient as possible. The leaf chamber temperature was set to the ambient temperature measured.

### Detailed screening in the growth chamber

On 9 April 2013, pots (ø, 16 cm; height, 50 cm) were filled with peat (Pindstrup Substrate no. 4, 10–30 mm, pH 6·0).

Rhizomes of each of the 11 genotypes from Denmark and Sin-7 from Sweden were dug up from the field on 16 April 2013. Sin-6 and Gig-14 were dug up on 11 March 2013 in Poland and stored at 2 ºC. Young rhizome pieces with live buds were planted in individual pots on 18 April. They were grown in the greenhouse for 7 weeks. The temperature in the greenhouse was set at 22/15 ºC day/night, the daylength was ambient and no supplementary light was given.

The plants were irrigated once or twice a week when necessary. A nutrient solution with 1 % inorganic fertilizer (Prima Væksthusgødning, NPK 3-1-4) was added on 8 May 2013 (300 mL per pot).

On 30 May, all plants were moved into a climate chamber and randomly arranged on trolleys with four replicates of each genotype. The plants were re-arranged randomly every second or third day in order to avoid the impact of position effects within the chamber. They were initially grown in warm conditions of 24/20°C and 14/10 h day/night cycles under a photon flux of 670 μmol m^–2^ s^–1^. The RH was set at 85 % day/night. After 10 d of acclimatization in warm conditions, light and CO_2_ response curve measurements were initiated as described below on a selection of 11 genotypes comprising one *M. tinctorius*, four *M. sinensis*, four *M. sacchariflorus* and two *M.×giganteus*.

After 22 d in warm conditions, the temperature was reduced to 14/10°C day/night on 24 June (designated Day 1 in cold conditions) with the same day/night cycle and light conditions as for the warm conditions. The RH under the cold conditions was 75/85 % day/night. All other conditions were set as described above. The measurement of light and CO_2_ response curves on the above-mentioned 11 genotypes in cold conditions was started on 30 June, which was Day 7 after the temperature had been decreased.

During the period of measurements in the climate chamber, the plants were irrigated every evening, and fertilizer was added every second or third day. ‘Nitaman 235’, which contains manganese at a concentration of 235 g L^–1^ and nitrate N at 120 g L^–1^, was sprayed on the leaves on 27 June due to manganese deficiency symptoms on leaves (leaves turned pale green in color).

### Gas exchange measurements and photosynthetic response curves

The carbon assimilation rate at a PAR of 1000 μmol m^–2^ s^–1^ (*A*_1000_) was measured once at the end of the warm period (designated Day 0), then 7 h after the temperature was decreased to 14°C (Day 1), and thereafter on Day 2, 3, 4, 5, 6, 9, 11, 15 and 18. A recovery measurement of *A*_1000_ was made on Day 24, equivalent to 36 h after the temperature was increased to 24/20°C day/night.

Gas exchange was always measured on the youngest fully developed leaf (ligule present) of all plants from 0830 to 1500 h, using an open-flow gas exchange system (CIRAS-2). The environment in the leaf cuvette was set in accordance with the climate chamber conditions, i.e. the CO_2_ concentration was set to 400 μmol mol^–1^ and mean leaf temperature was maintained at the experimental temperature (24 or 14°C). The vapour pressure deficit (VPD) in the leaf cuvette was kept at 1·2 kPa (warm conditions) and 1·0 kPa (cold conditions) by controlling the relative humidity, and the airflow through the chamber was 250 mL min^–1^.

For the light response curve measurements, the leaves were acclimatized in the leaf cuvette to a PAR of 2000 μmol m^–2^ s^–1^ until the photosynthetic rate stabilized (Wang *et al.*, 2012). Then the PAR was decreased from 2000 to 20 μmol m^–2^ s^–1^ in 14 steps (2000, 1800, 1500, 1200, 1000, 800, 500, 300, 200, 150, 100, 80, 50 and 20). The measurements were logged after photosynthetic rates had reached steady state. The rate of photosynthesis at a PAR of 1500 μmol m^–2^ s^–1^ (*A*_1500_) was defined as the saturated net photosynthetic rate (*A*_sat_).

For the determination of *A*–*C_i_* curves, leaves were acclimatized in the cuvette for 10 min at a CO_2_ concentration of 400 μmol mol^–1^ and a of PAR 1500 μmol m^–2^ s^–1^ until a steady state of *A* was reached. The CO_2_ concentration in the cuvette was decreased in eight steps (300, 250, 200, 150, 100, 80, 50 and 20 μmol mol^–1^ CO_2_) with around 5 min for each step. The CO_2_ concentration was then returned to 400 μmol mol^–1^ and kept at this level for about 10 min for stabilization. Thereafter it was increased in three steps (600, 1000 and 1200 μmol mol^–1^ CO_2_) with approx. 5 min for acclimatization at each step.

### Chlorophyll fluorescence measurements

Chlorophyll fluorescence was determined on Day 6 after the temperature had been reduced to 14°C, using a Mini-PAM fluorometer (Walz, Germany). The leaves were dark-adapted with dark leaf clips (Walz, Germany) for 30 min before the measurements were performed. The minimal fluorescence (*F*_0_) was determined at very low PAR, where the PSII reaction centres are in the ‘open’ state. The maximal fluorescence (*F*_m_) was measured by applying a 0·8 s pulse at a high light level of approx. 4000 μmol m^–2^ s^–1^, which drives the reaction centre to close (Krause and Somersalo, 1989). The maximum quantum yield of PSII (*F*_v_/*F*_m_) was calculated from *F*_0_ and *F*_m_ as *F*_v_/*F*_m_ = (*F*_m_ – *F*_0_)/*F*_m_.

### Specific leaf area (SLA)

Ten leaf discs with an area of 0·44 cm^2^ were cut from the first two fully expanded leaves (normally the third and fourth leaf counting from the top) using a cork borer with a diameter of 7·5 mm from the upper canopy of each plant after the gas-exchange measurements had finished. Leaf discs were oven-dried at 80°C for 3 d, weighed, and the SLA, which describes the ratio of leaf area to leaf dry matter (DM), was calculated as m^2^ kg^–1^. In addition, *A*_mass_ was calculated as *A*_sat_ multiplied by SLA.

### Photosynthetic response curves and calculation

Data from the light response experiment were fitted to a non-rectangular hyperbola model (Marshall and Biscoe, 1980) by means of the non-linear least squares curve-fitting procedure of R Studio for Windows (R Core Team, 2005).

The apparent quantum yield (AQY) values were obtained from linear regression of the relationship between net CO_2_ assimilation and PAR across five points at incident light intensities from 20 to 150 μmol m^–2^ s^–1^ (Long *et al.*, 1996). The dark respiration (*R*_d_) was estimated from the *y*-intercept of the initial linear line of five measured data points (Herrick and Thomas, 1999). *A*_sat_ was the rate of photosynthesis at a PAR of 1500 μmol m^–2^ s^–1^ (*A*_1500_).

In order to describe the response of *A* to *C*_i_, a linear regression was fitted for the initial linear part of the *A*–*C*_i_ curve (*C*_i_ <100 μmol mol^–1^) with the slope representing the carboxylation efficiency (CE) of PEPc. The CO_2_-saturated photosynthetic rate (*V*_max_) was calculated from the horizontal asymptote of the *A*–*C*_i_ curve (von Caemmerer, 2000). Stomatal limitation (Ls) was calculated from the *A*–*C*_i_ curves [Ls = (*A_C_*_i_ – *A_C_*_a_)/*A_C_*_i_; *C*_i_,*C*_a_ = 400 μmol mol^–1^] giving the photosynthetic reduction due to stomatal closure (Long and Bernacchi, 2003).

### Statistical analysis

Data for each temperature treatment were analysed separately using analysis of variance. If a significant effect was observed, multiple comparisons were performed using Turkey’s HSD (honest significant difference) test. All the analyses and tests were done in R version 3.1.2 (R Core Team, 2005). The R package ‘lsmeans’ (Lenth and Hervé, 2005) was used for multiple comparisons.

## RESULTS

### Ploidy

All *M. sacchariflorus* genotypes in this study were tetraploid, while all *M. sinensis* genotypes were diploid, and the two *M.* × *giganteus* genotypes were triploid and diploid, respectively (Table 1).

### Shoot growth and gas exchange in the field

For the 166 genotypes tested under field conditions in 2012, the daily shoot growth rate varied from 1·8 to 6·7 cm d^–1^ at high temperatures and from 0·7 to 3·6 cm d^–1^ at low temperatures (Fig. S1). The preliminary measurements of net photosynthesis in the field indicated a positive correlation between this variable and shoot growth rate at both high (*P* = 0·053) and low temperatures (*P* = 0·167) (Fig. S1) On the basis of these results, a total of 14 genotypes were selected for further investigation in the climate chamber in 2013. They were selected based on one or more of the following traits: high daily shoot growth rate during the cool period, high carbon assimilation rate during the cool period or late onset of flowering and senescence as observed in the field (data not shown). As we wanted to cover a broad variation in productivity, we also included genotypes with low daily shoot growth rates in cool conditions. Four miscanthus species were represented by the selection.

### Photosynthetic performance under warm and cold conditions

**Fig. 1.**
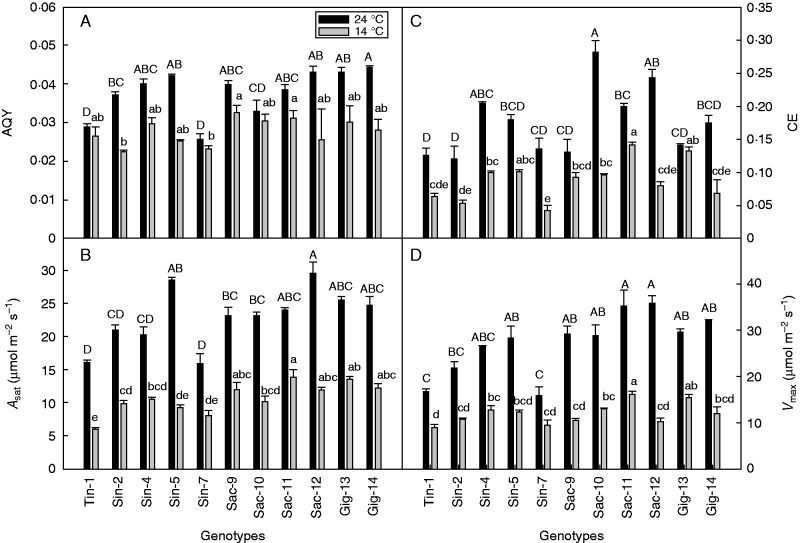
(A) Apparent quantum yield of CO_2_ uptake (AQY), (B) net photosynthetic rate at a PAR of 1500 μmol m^–2^ s^–1^ (*A*_1500_) of 11 miscanthus genotypes under warm (24 °C) and cold (14 °C) conditions; (C) carboxylation efficiency (CE) of phosphoenolpyruvate carboxylase (PEPc) and (D) maximum photosynthetic rate on the *A*–*C*_i_ curve (*V*_max_) of 11 miscanthus genotypes under warm (24 °C) and cold (14 °C) conditions. The CE was calculated as the slope of the net photosynthetic rate (*A*) vs. the intercellular CO_2_ concentration (*C*_i_) between 10 and 100 μmol m^–2^ s^–1^, and *V*_max_ as the asymptote of the *A****–****C*_i_ curve. Error bars represent the s.e. (*n* = 3 or 4). Values with the same letter are not significantly different at the *P* = 0·05 level; upper case letters show the comparison for the warm treatment and lower case letters for the cold treatment.

When the 14 selected genotypes were grown at the high temperature (24/20°C) they all displayed higher photosynthetic rates at all the measured light levels than they did at 14/10°C (data not shown). Furthermore, there were significant differences between genotypes under light-saturated conditions at both temperatures (Fig. 1C).

Most genotypes had higher AQY values when they were grown in warm conditions than under cold conditions (Fig. 1A). Under warm conditions, the genotypes Gig-14, Sac-12, Gig-13 and Sin-5 showed values of 0·045, 0·043, 0·043 and 0·042 mol mol^–1^, respectively, which were significantly higher than the values for Sac-10, Tin-1 and Sin-7 of 0·033, 0·029 and 0·023 mol mol^–1^, respectively. When the temperature was lowered to 14/10°C, Sac-9 and Sac-11 had significantly higher AQY values than Sin-2 and Sin-7, and the reduction in AQY was only 18 and 19 % in the two *M. sacchariflorus* genotypes; the reduction in Tin-1 and Sac-10 was even less.

The *R*_d_ was significantly higher under high temperatures, with the mean value of *R*_d_ declining from 1·94 to 0·91 μmol m^–2^ s^–1^ under cold conditions (data not shown). The genotypes Gig-14, Sac-12, Sac-11 had significantly higher rates of *R*_d_ than Sin-7 under warm conditions. Sac-9 showed a significantly higher *R*_d_ rate than Tin-1 at 14°C (data not shown). For Sin-7 and Tin-1, both AQY and *R*_d_ remained relatively stable when the temperature was lowered from 24 to 14°C. The rate of *R*_d_ accounted for 5·2–9·5 % of gross photosynthesis under warm conditions and 6·5–8·9 % under cold conditions, thus contributing only a minor share to gross photosynthesis. No significant difference was observed between genotypes concerning the light compensation point (LCP), with a mean value of 51 mol mol^–1^ at 24°C and 33 mol mol^–1^ at 14°C (data not shown).

The low growth temperature led to a significant reduction in *A*_sat_ (Fig. 1B). Sac-12 had the highest *A*_sat_ rate of the tested genotypes at 24°C at 29·5 μmol m^–2^ s^–1^, which fell to 11·9 μmol m^–2^ s^–1^ at 14°C. However, at this temperature Sac-11 had a significantly higher *A*_sat_ value (13·8 μmol m^–2^ s^–1^) than Sin-2, Sin-5, Sin-7 and Tin-1 (Fig. 1B). The largest reduction in *A*_sat_ with the temperature decrease was observed for Sin-5 (67·2 %) with a fall from 28·6 to 9·3 μmol m^–2^ s^–1^. Sin-4, Sac-9, Sac-11 and Gig-13 had the smallest reduction in *A*_sat_ (Fig. 1B). At 1500 μmol m^–2^ s^–1^ PAR, *C*_i_ increased for all genotypes when the temperature was decreased (data not shown), and Ls only accounted for 4–5 % of total limitation at 14°C, which indicates that non-stomatal limitation was the main reason for photosynthetic reduction.

Sac-10 and Sac-12 had significantly higher CE values than Gig-13, Sin-7, Tin-1, Sac-9 and Sin-2 at 24°C (Fig. 1C), and Sac-11 and Sac-12 had significantly higher *V*_max_ values than Sin-2, Tin-1 and Sin-7. When the temperature was decreased, the CE of PEPc decreased for all the genotypes, except for Gig-13 (0·14 at 24°C and 0·13 at 14°C). The maximum photosynthetic rate at saturated light and CO_2_ conditions was decreased by cold conditions in all genotypes (Fig. 1D). At 14°C, Sac-11 showed the highest values of CE and *V*_max_, but those of Gig-13 were not significantly lower (Fig. 1C, D).

Dark-adapted chlorophyll fluorescence (*F*_v_/*F*_m_) ranged from 0·52 to 0·75 at 14°C. Tin-1 showed significantly lower values than the other genotypes, except Sin-4, Sac-10, Sac-12 and Gig-14 (Supplementary Data Table S1).

### Development of *A*_1000_ in cold conditions and recovery of *A*_1000_ after 23 d in cold conditions

**Fig. 2.**
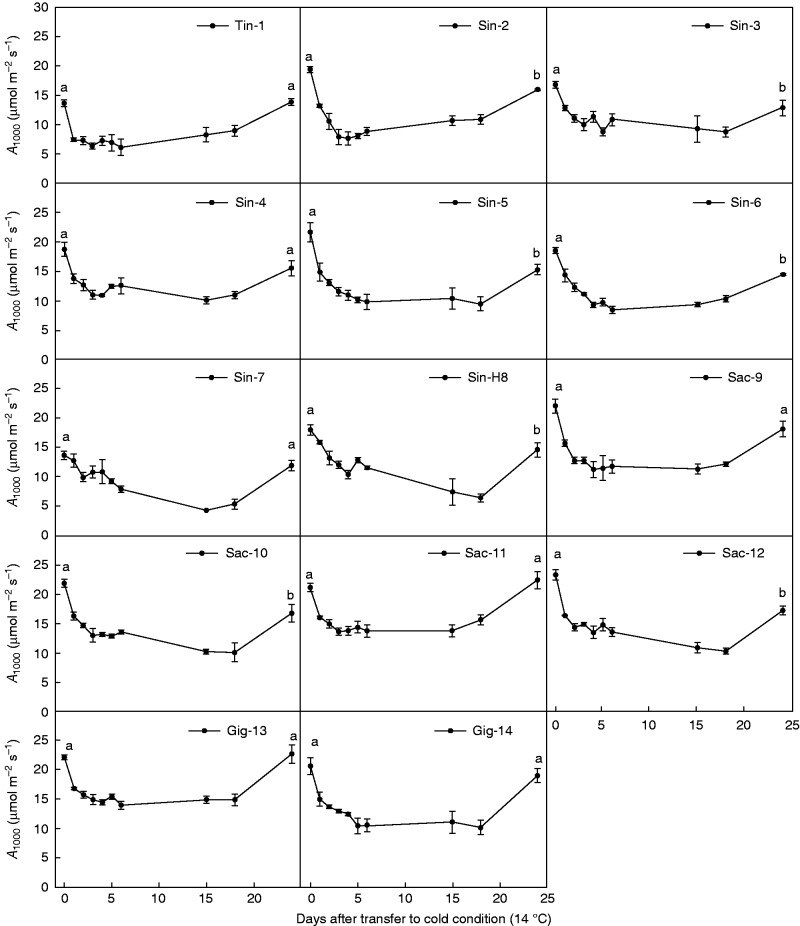
Development of the net photosynthetic rate at a PAR of 1000 μmol m^–2^ s^–1^ (*A*_1000_) of all genotypes before and after transfer of plants from warm conditions (24 °C) to cold conditions (14 °C), and 1 d after the temperature was returned to 24 °C. Error bars represent the s.e. (*n* = 3). Significant differences between genotypes on Day 0 before the temperature was lowered and on Day 24 after the temperature was increased were examined using the Student’s *t*-test. Values with the same letter are not significantly different at the *P* = 0·05 level.

A sharp reduction in the net photosynthetic rate was observed at 1000 μmol m^–2^ s^–1^ (*A*_1000_) for all genotypes 7 h after the temperature had been lowered from 24 to 14°C (Fig. 2). When compared with Day 1 at 14°C, *A*_1000_ was further reduced by < 20 % on Day 2 for most genotypes (Fig. 2). After Day 4, *A*_1000_ stabilized at a reduced level for most genotypes, while a few genotypes continued to decrease. For genotypes Tin-1, Sin-4, Sac-11 and Gig-1, *A*_1000_ fell by < 20 % after the initial drop measured after 7 h. The *A*_1000_ rates were significantly higher for Sac-11 and Gig-13 for most of the period (Fig. 2).

On Day 24, 1 d after the temperature had again been raised, *A*_1000_ increased for all genotypes. However, Sin-2, Sin-3, Sin-5, Sin-6, Sin-H8, Sac-10 and Sac-12 had significantly lower net assimilation rates than before the temperature drop, while the rest of the genotypes had values that were not significantly different from their pre-chilling values (Fig. 2). The Tin-1 and Sin-7 genotypes had the lowest values for all measurement dates.

### Specific leaf area

**Fig. 3.**
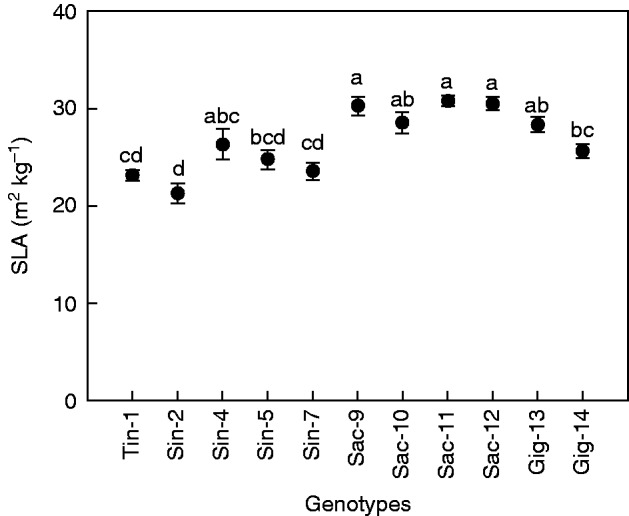
Specific leaf area (SLA) of the selected genotypes. Error bars represent the s.e. (*n* = 6). Values with the same letter are not significantly different at the *P* = 0·05 level.

**Fig. 4.**
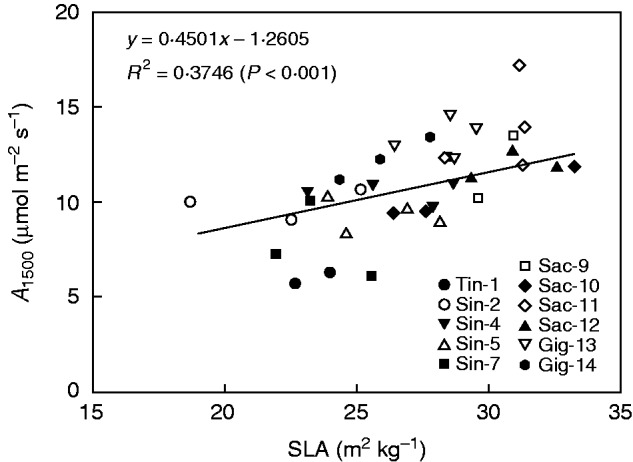
The linear relationship between the net photosynthetic rate at a PAR of 1500 μmol m^–2^ s^–1^ (*A*_1500_) and specific leaf area (SLA) under cold conditions. Each point is the mean of SLA from each plant and its correlated *A*_1500_ from the same replication.

Overall, the *M. sacchariflorus* species had higher SLAs than *M. sinensis* and *M. tinctorius* (Fig. 3). The Sac-11 genotype had the highest SLA at 30·7 m^2^ kg^–1^ and Sin-2 the lowest at 21·3 m^2^ kg^–1^. Sac-11, Sac-12 and Sac-9 had significantly higher SLAs than Tin-1, Gig-14 and all the *sinensis* genotypes, except for Sin-4 (Fig. 3). *A*_sat_ showed a positive linear correlation with SLA at the low temperature (Fig. 4), and the relationship between SLA and *A*_mass_ was also linear and positive, with an *R*^2^ value of 0·68 (Supplementary Data Fig. S2).

### Relationship between daily shoot growth rate and *A*_1000_ under cold conditions

**Fig. 5.**
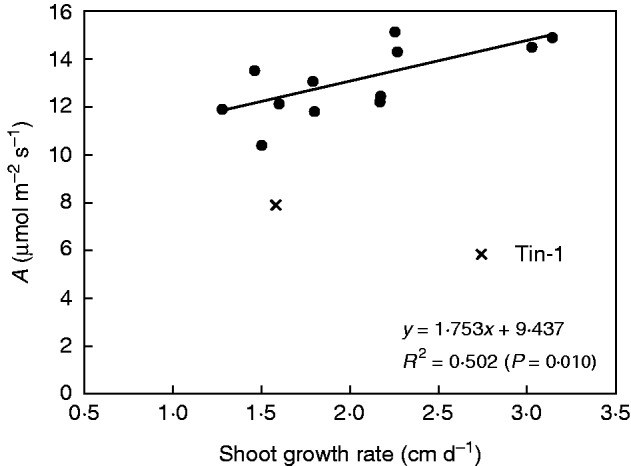
Relationship between shoot growth rate measured under cool 9·8/15·5 °C (mean/maximum) conditions in the field in 2012 and mean photosynthetic rate at 1000 μmol m^–2^ s^–1^ (mean *A*_1000_) measured for 13 genotypes (Tin-1 is excluded in the relationship but shown on the graph) 3 d after the temperature was decreased from 24 to 14 °C in the climate chamber in 2013.

Of the genotypes selected for the detailed study, Sac-12 and Sac-11 had the highest shoot growth rates measured at low temperatures under field conditions in Denmark in 2012 (data not shown). A positive linear relationship with an *R*^2^ value of 0·502 (*P* = 0·01) was found between daily shoot growth rate in the field and average values of *A*_1000_ measured on *M. sinensis*, *M. sacchariflorus* and *M.* × *giganteus* (Tin-1 was excluded from this analysis as it did not fit the same relationship as the other species) 3 d after the temperature decrease in the growth chambers in 2013 (Fig. 5).

## DISCUSSION

### Cold tolerance under cold conditions

We found that the photosynthetic parameters of *M.* × *giganteus* ‘Hornum’ (Gig-13) were very competitive compared with those of other miscanthus genotypes selected from the germplasm collection of 166 individuals, and it showed the lowest reduction in *A*_sat_ when the temperature was decreased from 24 to 14°C. This is in agreement with previous findings that, of the C_4_ grasses, the widely investigated triploid *M.* × *giganteus* is exceptionally productive in cold climates and has the ability to maintain high photosynthetic activity at 14°C (Naidu *et al.*, 2003). However, one *M. sacchariflorus* (genotype Sac-11) showed slightly higher values of AQY, *A*_sat_, CE and *V*_max_ than Gig-13, even though it was not significantly different at 14°C. At 24°C, two *M. sacchariflorus* genotypes (Sac-10 and Sac-12) showed significantly higher CE values than *M.* × *giganteus*. Głowacka *et al.* (2015*b*) screened the plants of the same germplasm collection with the least frost damage in the field with a different procedure (chlorophyll fluorescence). Sac-10 was the only genotype that could be compared directly between our study and that of Głowacka *et al.* (2015*b*) (where it was named Msa ‘44/1’). We measured the same level of *A*_1500_ in sac-10 in the two studies. However, Głowacka *et al.* (2015*b*) found two *M. sacchariflorus* genotypes that had significantly higher photosynthetic capacity than *M.* × *giganteus* at 15°C, with a higher *A*_1500_ level than any genotype selected in our study. This may indicate that the two-stage procedure (first a field frost event and then *F*_v_/*F*_m_ screening of the surviving genotypes) is a particularly efficient method for field screening for cold tolerance, which, however, is difficult to replicate on another population. The linear part of the light response curve reflects the maximum efficiency of light harvesting, and a reduction in the AQY is associated with photoinhibition (Long *et al.*, 1994). This can be due to increased non-photochemical energy dissipation as a photoprotective mechanism or to photodamage to the reaction centre of PSII (Osmond, 1994).

The genotypes Sac-9 and Sac-11 had the highest AQY values at 14°C and the lowest reduction in AQY when the temperature was decreased from 24 to 14°C. In northern Europe, saturating light is difficult to reach in the field, and light-limited photosynthesis contributes significantly to canopy carbon gain (Baker *et al.*, 1988). Therefore, a high AQY has greater influence on photosynthetic capacity than a high *A*_sat_ under light-limited conditions and/or within a closed canopy (Long, 1983). Even though values of AQY in Sac-9 and Sac-11 were not significantly higher than the values of Gig-13, the *F*_v_/*F*_m_ values in Sac-9 and Sac-11 were slightly higher (not significant) than that of Gig-13. *F*_v_/*F*_m_ is a measure of the maximum quantum efficiency of PSII, and a decrease is also indicative of photoinhibition (Long *et al.*, 1994; Maxwell and Johnson, 2000). Furthermore, the slight decrease in photosynthetic performance of Sac-9 and Sac-11 as well as of Gig-13 was reversible within a day, as confirmed by the recovery of *A*_1000_ after increasing the temperature from 14 to 24°C (Fig. 2). All these results indicate that it is possible to find suitable genotypes for breeding of new improved triploid *M*. × *giganteus* varieties, and the two tetraploid *M. sacchariflorus* genotypes seem to be good candidates for achieving high productivity in light-limited environments at low temperature.

The lower photosynthetic activity at low temperatures has previously been found in *M*. × *giganteus* not to be due to stomatal limitations (Naidu and Long, 2004; Farage *et al.*, 2006), and our results of the Ls of the 11 genotypes confirmed this.

The key enzymes associated with carbon fixation, namely PPDK (Naidu *et al.*, 2003, Wang *et al.*, 2008*b*) and Rubisco (Spence, 2012), are sensitive to low-temperature damage in *M*. × *giganteus* and are thus central to maintaining high photosynthetic activity at low temperature. *V*_max_ is controlled by a variety of processes, mainly PEP regeneration via the key enzymes mentioned above (Collatz *et al.*, 1992; von Caemmerer, 2000). Sac-11 and Gig-13, with their significantly higher *A*_sat_, both had higher *F*_v_/*F*_m_ values and a lower reduction in AQY than several other genotypes at 14°C. The significantly higher *V*_max_ values in Sac-11 and Gig-13 than in several other genotypes are indicative of relatively high amounts or activity of the key enzymes involved in carbon fixation at the low temperature.

At low *C*_i_, the CE of PEPc controls the photosynthetic rate (Collatz *et al.*, 1992; von Caemmerer, 2000). There was a slight but insignificant decrease in CE values in Gig-13 when the temperature was decreased, which is similar to previous findings (Naidu and Long, 2004). Sac-11 had slightly higher CE values at both temperatures than Gig-13 (but not significant at 14°C), whereas in all the other genotypes CE decreased significantly when the temperature was lowered from 24 to 14°C. This suggests that the amount and activity of PEPc might be a limiting factor at low temperatures for most miscanthus genotypes, with *M*. × *giganteus* and Sac-11 as exceptions.

It is interesting to note that most *M. sacchariflorus* genotypes had higher *A*_sat_ values than the *M. sinensis* genotypes at 14°C (Fig. 1B). This is in agreement with previous findings that *M. sacchariflorus* typically has a higher tolerance to chilling than *M. sinensis* (Głowacka *et al.*, 2014). This is also evident from the natural geographical distribution of *M. sacchariflorus* which extends further north in Asia than *M. sinensis* (Clifton-Brown *et al.*, 2008).

### Development of *A*_1000_ under cold conditions and recovery after re-increase of the temperatures

The development patterns differed within the miscanthus genotypes and they indicate that there are different reactions during cold temperature exposure.

The reduction in *A*_1000_ for Gig-13 and Sac-11 ranged from 12 to 20 % compared with the value on Day 1 during the 18 d of cold treatment. Previous studies detected a drop in *A*_1000_ in *M*. × *giganteus* immediately after the change from 24 to 14°C, but after 3 d a recovery was observed and the reduction was only 12 % on the ninth day (Wang *et al.*, 2008*b*). However, in most other genotypes, we observed a continued decrease in *A*_1000_ after Day 4, which could be due to a further decrease in the amount and activity of PPDK and/or Rubisco (as observed in maize during prolonged cold conditions; Wang *et al.*, 2008*b*), but perhaps also caused by an accumulation of xanthophyll (violaxanthin, antherananthin and zeaxanthin) cycle pigments, which apparently is a cold tolerance strategy for C_4_ species whereby they dissipate surplus energy (Kubien and Sage, 2004).

A previous study has shown that the rapid recovery of *M.* × *giganteus* photosynthesis at 14/11°C due to the temperature response of light-saturated photosynthesis is very similar for *M.* × *giganteus* grown in warm (25/20°C) and cold (14/11°C) conditions (Naidu *et al.*, 2003). In our study, genotypes Sin-2, Sin-3, Sin-5, Sin-6, Sin-H8, Sac-10 and Sac-12 did not have the capacity to recover their photosynthesis rapidly after a long cold period. Only the genotype Tin-1 showed a significantly lower *F*_v_/*F*_m_ than the other genotypes, indicating a loss of PSII capacity, while Sin-3, Sin-5, Sin-6 and Sin-H8 showed relatively high *F*_v_/*F*_m_ values at 14°C (Table S1), so the failure of a quick recovery may be explained rather by the high xanthophyll levels that continued to dissipate absorbed light and/or to reduced contents of PPDK and/or Rubisco. For the genotypes Sac-11and Gig-13, *A*_1000_ recovered to a similar level to that before the cold treatment after a 24 h exposure to the warm temperature, which indicates that a temperature of 14°C did not cause irreversible photoinhibition in PSII, and that these two genotypes are more suited to variable, cool, temperate conditions because of their high net photosynthetic rate under both light-limited and light-saturated conditions. Such reversible dynamic photoinhibition is apparently a strategy by which C_4_ species may tolerate the low temperatures that prevail in temperate climate zones (Kubien and Sage, 2004).

### Correlation between photosynthetic capacity and two morphological characters: SLA and shoot growth rate

In our study we observed that genotypes with a high SLA were capable of attaining even higher photosynthetic efficiency than genotypes with a low SLA under cold conditions (Fig. 4). This indicates that within the *Miscanthus* genus there is significant variation in the photosynthetic return of investment in leaf biomass, which is an important trait for high productivity early in the season. The SLA of miscanthus has in previous studies been significantly higher than for switchgrass (*Panicum virgatum*) and had significantly higher *A* values than for switchgrass (Dohleman *et al.*, 2009). This shows that having thinner leaves is not necessarily at the expense of a low *A*. The significantly higher SLA in Sac-11 and Gig-13 combined with their high cold tolerance hints that these two genotypes have the ability to develop a large leaf canopy quickly during the early spring in maritime climates.

The relationship between SLA and net photosynthesis per unit leaf area has been rarely investigated (Reich *et al.*, 1998). In our study, a positive correlation between SLA and *A* was confirmed. However, whether the leaf thickness is an adaptation to the cold conditions or an inherent characteristic within each genotype is not clear, and this should be further investigated.

Of the 14 genotypes tested in this study, we found significantly higher shoot growth rates for genotypes Sac-11 and Gig-13 in the field than for most other genotypes investigated in detail in climate chambers (data not shown). The genotypes Sac-11 and Gig-13 also had relatively high *A*_1000_ values during the first 3 d at 14°C. The good linear relationship between shoot growth rate and photosynthesis of the investigated genotypes (except *M. tinctorius*) provided another simple procedure for selecting genotypes from large-scale field collections of *M. sinensis*, *M. sacchariflorus* and *M.* × *giganteus* for further detailed investigation. The Tin-1 genotype had the lowest photosynthetic performance under both temperature treatments in this study. This result was also achieved by Głowacka *et al.* (2015*b*), where *M. tinctorius* had the lowest cold tolerance with the lowest photosynthetic rate compared with *M. sinensis*, *M. sacchariflorus* and *M.* × *giganteus.* The *M. tinctorius* genotypes grow quite well in the field in Denmark, and this species seems to have a different physiological response to cope with cold temperatures compared with the other miscanthus species.

## Conclusion

No severe cold stress was detected at 14°C in any of the genotypes selected. Although not significant, the genotype Sac-11 seemed to perform slightly better in terms of cold tolerance and quick recovery than the hitherto most widely used triploid *M*. × *giganteus* (Gig-13), plus its high SLA and shoot growth rate gave a fast leaf canopy development in the early growing season. Therefore, it may be used for large plantations together with *M*. × *giganteus* or for breeding new interspecies hybrids with improved traits for temperate climates. Sac-12 performed significantly better than *M*. × *giganteus* (Gig-13) at 24°C and high light intensities, and may be used for breeding improved varieties for warm climates. Interestingly, we found that two phenotypic variables that are relatively easy to measure, i.e. daily shoot growth rate and SLA, correlated well with leaf photosynthesis. These variables may be used for the selection of individuals from a large gene pool for more detailed analysis or for screening new hybrids. The ability of some genotypes to fix significantly more carbon per gram of leaf biomass than others and the discovery of a generally positive correlation between SLA and *A*_max_ were surprising compared with our hypothesis that *A*_max_ would remain unchanged at increased SLA. This leaf variable will be important for further studies and may be used for the selection of ideotype biomass crops with the most efficient solar capture and carbon fixation. Another interesting area for future investigation would be whether miscanthus genotypes become more disparate in their photosynthetic capacity at even lower temperatures.

## SUPPLEMENTARY DATA

Supplementary data are available online at www.aob.oxfordjournals.org and consist of the following. Table S1: maximum quantum yield of PSII (*F*_v_/*F*_m_) measured at 14°C. Figure S1: correlation between daily shoot growth rate and net photosynthesis in 37 miscanthus genotypes measured in field trials in Denmark in 2012 during a warm and a cold period. Figure S2: the linear relationship between *A*_mass_ and SLA in cold conditions.

Supplementary Data
