# Supplementary material for: Low-temperature leaf photosynthesis of a Miscanthus germplasm collection correlates positively to shoot growth rate and specific leaf area
Source: Ann Bot. 2016 May 13;117(7):1229–39. doi: 10.1093/aob/mcw042 (PMC4904170; doi:10.1093/aob/mcw042)
Supplement: Supplementary Data [file supp_117_7_1229__index.html]

Low-temperature leaf photosynthesis of a Miscanthus germplasm collection correlates positively to shoot growth rate and specific leaf area — Supplementary Data 

# Low-temperature leaf photosynthesis of a *Miscanthus* germplasm collection correlates positively to shoot growth rate and specific leaf area

## Supplementary Data

files

- Supplementary Data - pdf file
