# Supplementary material for: Low-temperature leaf photosynthesis of a Miscanthus germplasm collection correlates positively to shoot growth rate and specific leaf area
Source: Ann Bot. 2016 May 13;117(7):1229–39. doi: 10.1093/aob/mcw042 (PMC4904170; doi:10.1093/aob/mcw042)
Supplement: Supplementary Data [file supp_mcw042_mcw042suppdata.pdf]

SUPPLEMENTARY DATA

Table S1. Maximum quantum yield of PSII (F_v/F_m) measured at 14°C. Values with the same letter are not significantly different at the P = 0.05 level.

Identification	F_v/F_m (Mean ± SE)			
Tin-1	0.52	±	0.01	b
Sin-2	0.69	±	0.01	a
Sin-3	0.75	±	0.03	a
Sin-4	0.64	±	0.03	ab
Sin-5	0.73	±	0.01	a
Sin-6	0.72	±	0.04	a
Sin-7	0.71	±	0.02	a
Sin-H8	0.72	±	0.03	a
Sac-9	0.74	±	0.06	a
Sac-10	0.66	±	0.00	ab
Sac-11	0.74	±	0.01	a
Sac-12	0.66	±	0.02	ab
Gig-13	0.71	±	0.02	a
Gig-14	0.63	±	0.03	ab

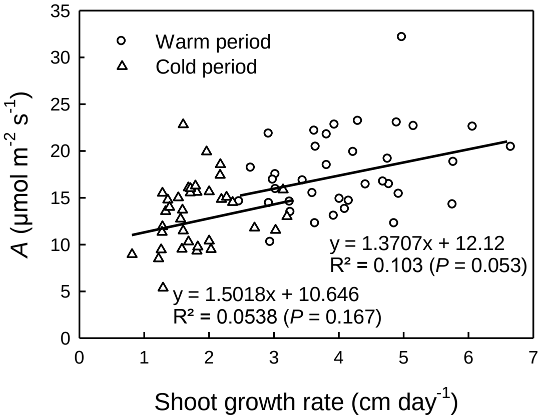

Figure S1. Correlation between daily shoot growth rate and net photosynthesis (A) in 37 *Miscanthus* genotypes measured in field trials in Denmark in 2012 during a warm (24–26 May, triangles) and cold (31 May to 1 June, squares) period. Legends marked in dark grey indicate genotypes that were selected for measurements in the climate chamber in 2013.

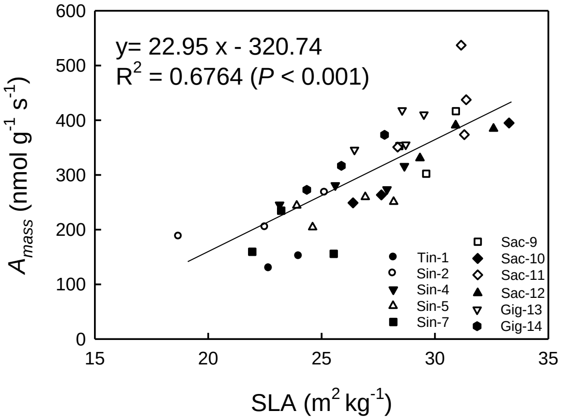

Figure S2. The linear relationship between A_{mass} and SLA in cold conditions. Each point represents one replication. Each point is the mean of SLA from each plant and its correlated A_{1500}.
